# PRMxAI: protein arginine methylation sites prediction based on amino acid spatial distribution using explainable artificial intelligence

**DOI:** 10.1186/s12859-023-05491-x

**Published:** 2023-10-04

**Authors:** Monika Khandelwal, Ranjeet Kumar Rout

**Affiliations:** Computer Science and Engineering Department, National Institute of Technology Srinagar, Hazratbal, Srinagar, J&K 190006 India

**Keywords:** Arginine methylation, Physicochemical properties, Shannon entropy, Machine learning algorithms, SHAP, Explainable AI

## Abstract

**Background:**

Protein methylation, a post-translational modification, is crucial in regulating various cellular functions. Arginine methylation is required to understand crucial biochemical activities and biological functions, like gene regulation, signal transduction, etc. However, some experimental methods, including Chip–Chip, mass spectrometry, and methylation-specific antibodies, exist for the prediction of methylated proteins. These experimental methods are expensive and tedious. As a result, computational methods based on machine learning play an efficient role in predicting arginine methylation sites.

**Results:**

In this research, a novel method called PRMxAI has been proposed to predict arginine methylation sites. The proposed PRMxAI extract sequence-based features, such as dipeptide composition, physicochemical properties, amino acid composition, and information theory-based features (Arimoto, Havrda-Charvat, Renyi, and Shannon entropy), to represent the protein sequences into numerical format. Various machine learning algorithms are implemented to select the better classifier, such as Decision trees, Naive Bayes, Random Forest, Support vector machines, and K-nearest neighbors. The random forest algorithm is selected as the underlying classifier for the PRMxAI model. The performance of PRMxAI is evaluated by employing 10-fold cross-validation, and it yields 87.17% and 90.40% accuracy on mono-methylarginine and di-methylarginine data sets, respectively. This research also examines the impact of various features on both data sets using explainable artificial intelligence.

**Conclusions:**

The proposed PRMxAI shows the effectiveness of the features for predicting arginine methylation sites. Additionally, the SHapley Additive exPlanation method is used to interpret the predictive mechanism of the proposed model. The results indicate that the proposed PRMxAI model outperforms other state-of-the-art predictors.

## Background

Protein methylation is a reversible procedure of post-translational modifications (PTMs) of proteins, and it may happen on arginine, proline, lysine, histidine, and carboxyl groups. Proteins play a significant role in an organism’s life and cellular processes. After the protein synthesis, further modifications can be needed to acquire functional and structural variation in the proteome. These modifications are known as PTMs. Protein methylation received less attention than other PTMs due to insufficient data [1, 2]. In protein methylation, proteins are enzymatically altered by adding methyl groups. Protein arginine methyltransferases (PRMT) carried out these additions by transferring a methyl group from S-adenosylmethionine. The other types of PTMs are phosphorylation [3], ubiquitination [4], sumoylation [5], acetylation [6], and N6-methyladenosine (m$^{6}$A) [7]. PTMs are necessary for driving various cellular processes, including gene transcription, RNA processing, signal transduction, regulation, and signaling pathways [8–10].

Recent research on methylation suggests that regulative enzymes are responsible for various human disorders, including multiple sclerosis, rheumatoid arthritis, coronary heart disease, neurodegenerative disorders, SARS virus, and cancer [11–14], due to their involvement in the regulation of gene expression. So, methylation sites should be recognized to comprehend the chemical structure of proteins better. Understanding the molecular mechanisms underlying protein methylation requires the capacity to recognize methylation sites. However, experimental techniques, including Chip–chip and mass spectrometry, are time-consuming and expensive [15–17]. As a result, computational methods based on artificial intelligence (AI) are needed to predict arginine methylation sites efficiently.

**Fig. 1:**
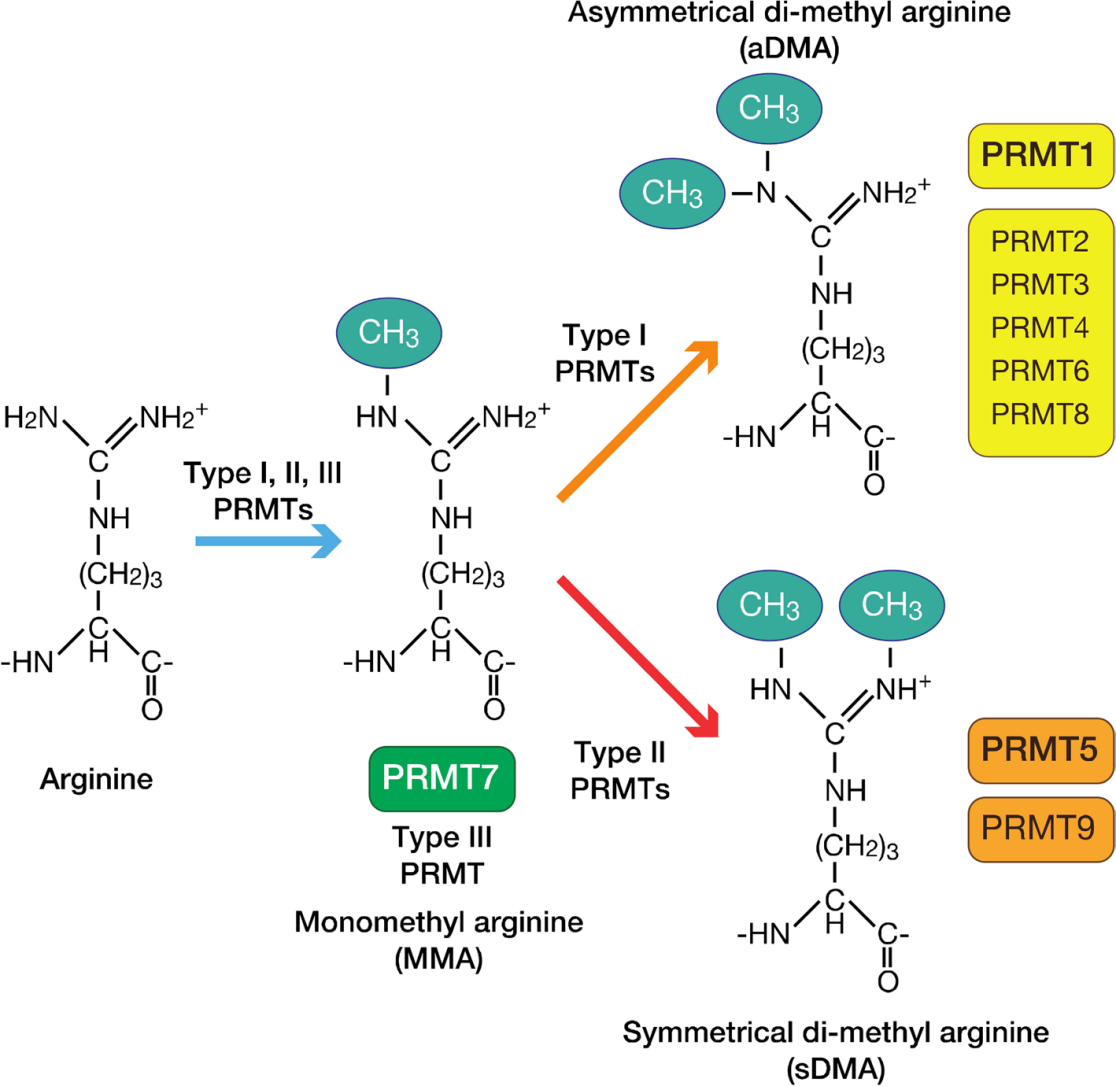
Three different types of arginine methylation: mono-methylarginine, asymmetric di-methylarginine, and symmetric di-methylarginine [21]

Protein methylation happens typically at the N-terminal side chain of arginine (R), which is the subject of this study due to their physicochemical and biological properties [18–20]. One or two methyl groups are attached to the nitrogen atom of arginine in the protein sequences during arginine methylation, as shown in Fig. 1 [21]. Three methylation forms are found in arginine: mono-methylarginine, asymmetric di-methylarginine, and symmetric di-methylarginine. It happens in glycine (G) and arginine (R) areas, impacting the interaction between proteins and structure. Arginine methylation is required in different cellular processes, such as cellular proliferation, genome stability, RNA processing, DNA repair, transcription regulation, signal transduction, and cancer [8, 22, 23].

Various artificial intelligence-based computational methods have been developed to recognize methylated sites in protein sequence data. These techniques deliver accurate, reliable, and faster calculations. Numerous other problems, such as protein classification, protein-protein interaction, etc. involve using machine learning methods [24–28]. Daily et al. [29] devised a strategy using the supervised learning method-support vector machines (SVM) to identify methylation sites based upon specific characteristics that gather disorder information from protein sequences. Subsequently, Chen et al. [30] devised MeMo to predict methylation sites based on SVM and orthogonal binary feature descriptors. The disadvantage of previous predictors was that they used an orthogonal binary encoding scheme to represent the primary sequence information and needed to consider structural information around the methylated sites. To overcome this, Shien et al. [31] devised a model named MASA that combines structural characteristics, i.e., secondary structure and accessible surface area, with sequence information. To further enhance the prediction quality, Qiu et al. [32] devised a model named iMethyl-PseAAC by integrating features, including sequential evolution, physicochemical, structural disorder knowledge, and amino acid composition, with SVM.

Furthermore, some researchers suggested extracting the primary sequence data using physicochemical properties, position weight amino acid composition, or sequential information [33, 34]. A sequence-based model called MePred-RF was proposed by Wei et al. [35] using a random forest (RF) algorithm. However, their benchmark data set consists of only 185 arginine sites and 226 lysine sites. Kumar et al. [36] proposed a prediction model named PRmePRed for arginine methylation based on structural and physicochemical properties using SVM. An arginine methylation prediction method, CTD-RF, developed by Hou et al. [37] that integrates RF with distribution, composition, and transition features. Some of the researchers also used convolutional neural network (CNN) and long short-term memory (LSTM) deep learning algorithms for the prediction of arginine methylation sites [38, 39].

Although the methods mentioned above have their own merits and have contributed to the growth of this field, they also possess some limitations and need enhancement in one or more of the below aspects: (1) most existing methods need evolutionary, disorder, and structural information for extracting features, which is not always available. Producing this kind of information depends on third-party computing software; the output of different software may vary. (2) The data set used to train existing methods is less than the current methylation sites. The existing methods’ data set must be updated by including new experimentally verified data. (3) Further, improving the predictive power using more informative features. By focusing on the above issues, we proposed a model named PRMxAI to identify arginine methylation sites using sequence information and the RF classifier. The main contributions of this research are as follows:

- The proposed model PRMxAI exploits sequence-based features, including physicochemical properties (PP), dipeptide composition (DPC), information theory-based characteristics (ITB), and amino acid composition (AAC).
- The performance of different classifiers (RF, decision tree (DT), k-nearest neighbors (KNN), Naive Bayes (NB), and SVM) are shown to select the better classifier to predict protein methylation sites.
- This research finds the effect of various features, i.e., PP, DPC, AAC, and ITB, on the arginine methylation data set.
- The proposed model interpretation is also shown using explainable AI (XAI).

**Fig. 2:**
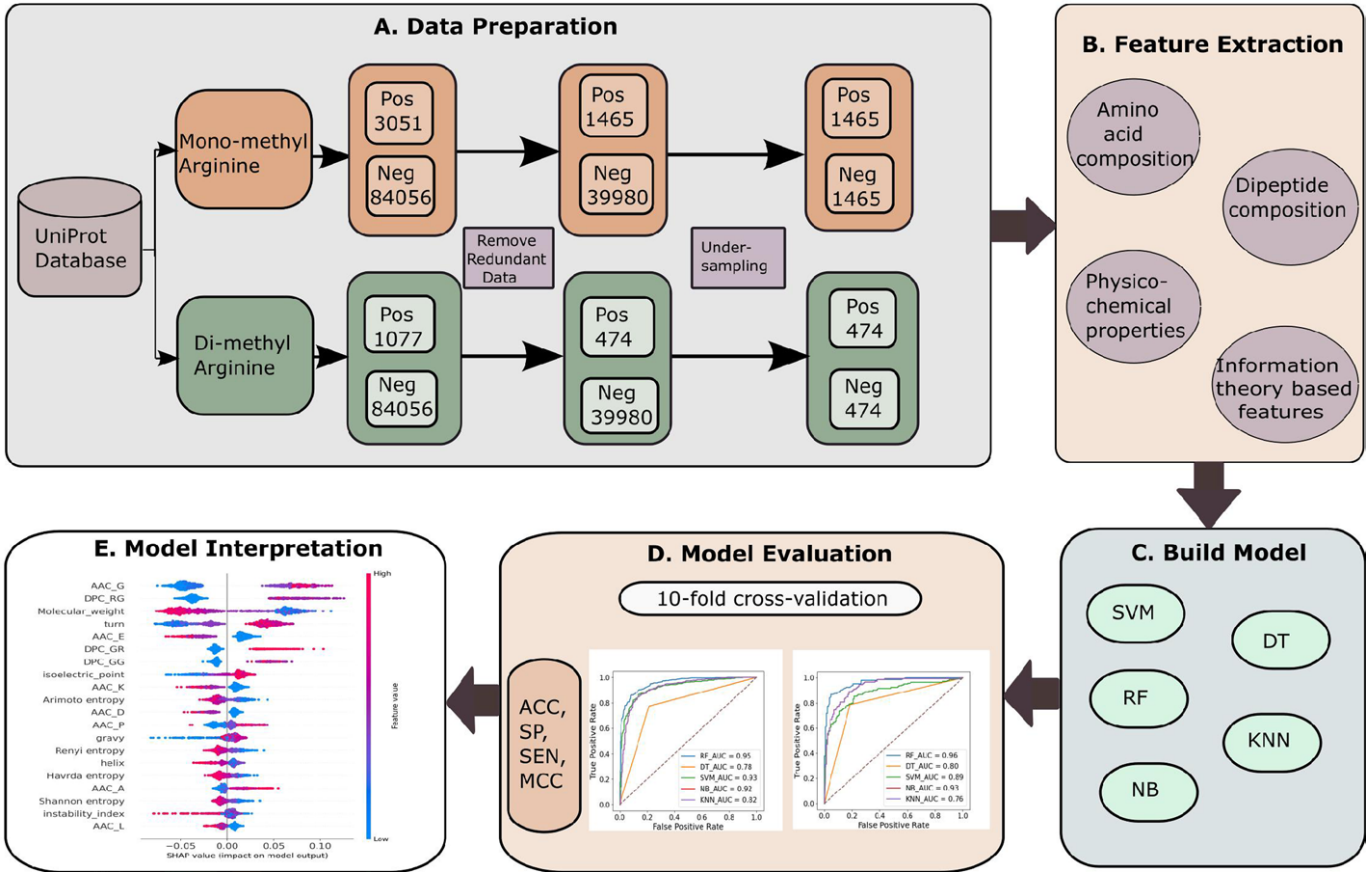
Architecture of the proposed model to predict arginine methylated sites. **a** Data collection, removal of redundant sequences, and data balancing using under-sampling. **b** Feature extraction including the dipeptide composition, physicochemical properties, amino acid composition, and information theory-based features. **c** Build a model for different machine learning algorithms. **d** Model evaluation using various evaluation parameters based on 10-fold cross-validation. **e** Model interpretation of model outputs using SHAP algorithm

The complete architecture of the proposed model is shown in Fig. 2.

## Results

This section found the performance of various classifiers to select the better-performing classifier to predict mono-methylarginine and di-methylarginine sites in protein sequences.

### Performance of various classifiers

**Fig. 3:**
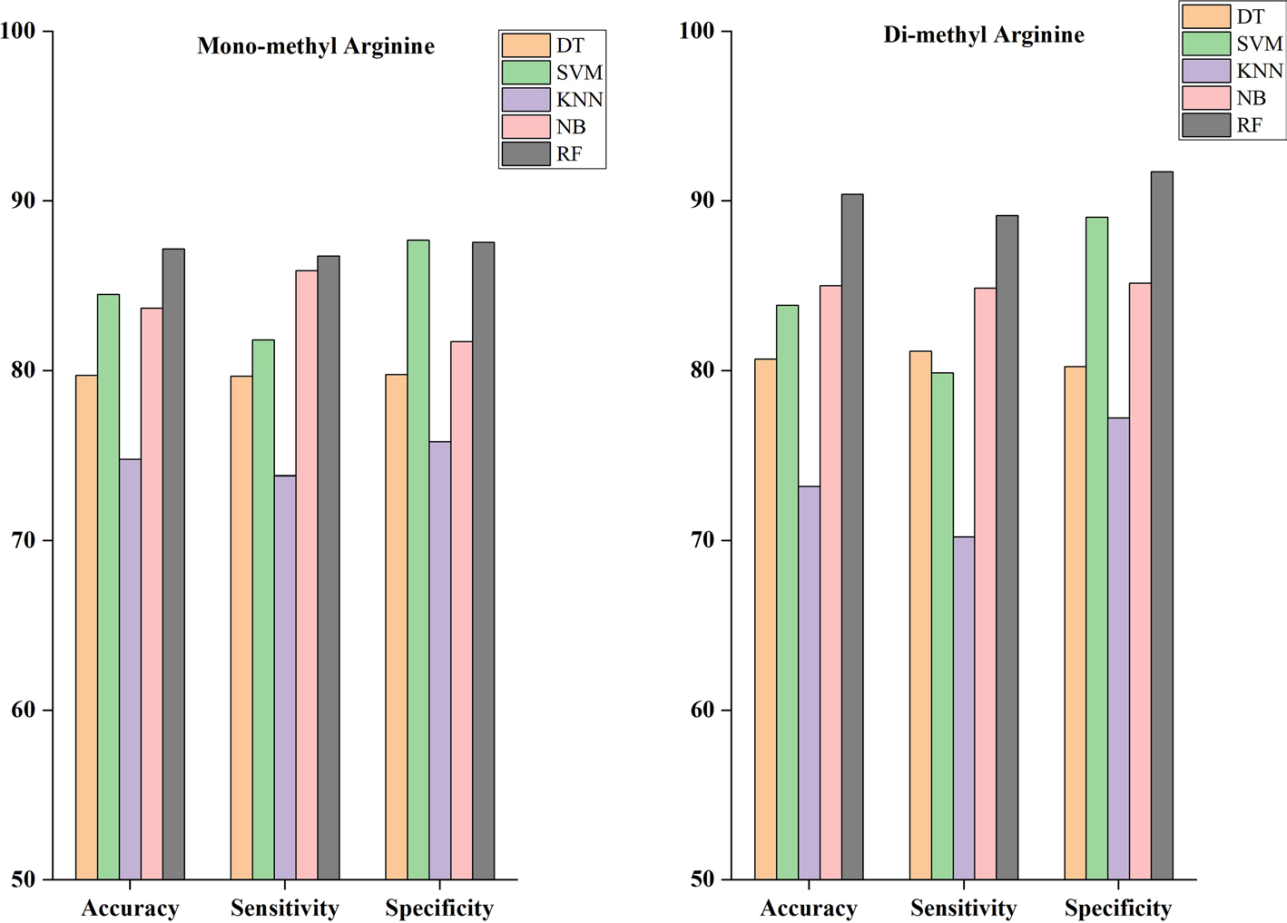
Performance of the KNN, SVM, RF, NB, and DT classifiers on the mono-methylarginine and di-methylarginine data sets

The feature representation includes ITB, DPC, PP, and AAC to find the performance of various classifiers. These features are essential in various ways when extracting information from peptide sequences. The problem is utilizing supervised machine learning algorithms to find meaningful patterns from the training data due to the varied significance of the extracted information. The widely used supervised machine learning algorithms are RF, NB, SVM, KNN, and DT. The various evaluation parameters, such as sensitivity (SEN), Matthew’s correlation coefficient (MCC), specificity (SP), and accuracy, are used to estimate the performance of various classifiers. The comparison of various classifiers for mono-methylarginine and di-methylarginine data sets are reported in Tables 1 and 2, respectively. Figure 3 demonstrates the predictive results of different algorithms to predict mono-methylarginine and di-methylarginine sites.

**Table 1:** Performance comparison of the various classifiers for mono-methylarginine data set

Classifiers	ACC (%)	SP (%)	SEN (%)	MCC	F1-score (%)	AUC
DT	79.73	79.77	79.69	0.59	79.84	0.78
SVM	84.51	87.69	81.81	0.69	85.10	0.93
KNN	74.78	75.82	73.82	0.49	75.27	0.82
NB	83.68	81.73	85.89	0.67	83.13	0.92
RF	87.17	87.58	86.76	0.74	87.14	0.95

**Table 2:** Performance comparison of the various classifiers for di-methylarginine data set

Classifiers	ACC (%)	SP (%)	SEN (%)	MCC	F1-score (%)	AUC
DT	80.69	80.24	81.15	0.61	80.57	0.80
SVM	83.86	89.05	79.88	0.68	84.85	0.89
KNN	73.20	77.22	70.22	0.46	75.06	0.76
NB	85.02	85.16	84.87	0.70	84.40	0.93
RF	90.40	91.72	89.16	0.80	90.54	0.96

**Fig. 4:**
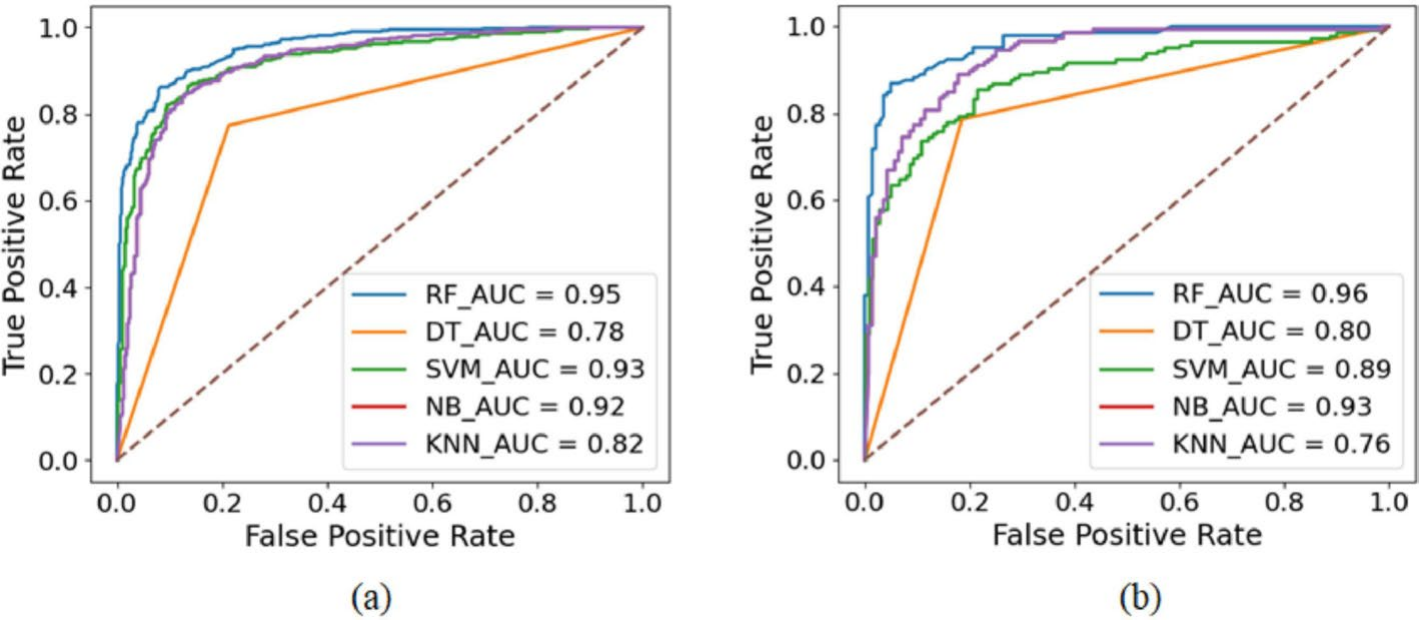
Area under ROC curve (AUC) for various classifiers **a** to predict mono-methylarginine sites. **b** to predict di-methylarginine sites

From Fig. 3, we notice that the RF outperforms other classifiers in accuracy, specificity, and sensitivity for predicting mono-methylarginine and di-methylarginine sites. Tables 1 and 2 show that the MCC is higher for the RF for predicting mono-methylarginine and di-methylarginine sites. The receiver operating characteristic (ROC) curve was shown to examine how well different classifiers performed. A ROC curve is plotted by drawing the actual positive rate versus the false positive rate. Figure 4a, b show the area under the ROC curve (AUC) for mono-methylarginine and di-methylarginine sites, respectively. The AUC for predicting mono-methylarginine and di-methylarginine sites is more significant when using the RF algorithm. Therefore, the RF is the underlying classifier for predicting arginine methylated sites from primary sequences.

### Proposed model performance

**Fig. 5:**
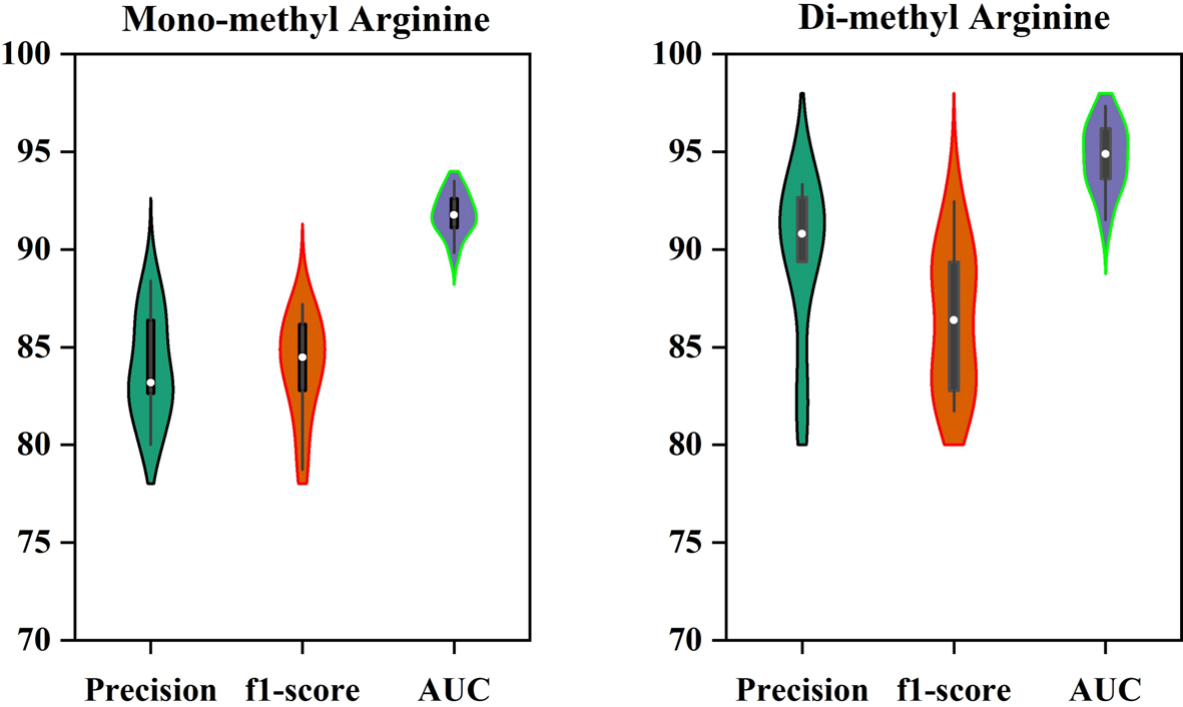
Performance of the proposed model for the mono-methylarginine and di-methylarginine data sets

The performance of the PRMxAI has been evaluated for mono-methylarginine and di-methylarginine data sets. The supervised learning algorithm RF is utilized to train the model after finding the features from amino acid sequences, and the learned model is then applied to generate predictions. The features used include DPC, PP, AAC, and ITB. A 434-dimensional vector characterizes each peptide sequence. We analyze the performance of the PRMxAI employing 10-fold cross-validation. For the mono-methylarginine data set, the proposed model yields 87.17% accuracy, 87.58% specificity, 86.76% sensitivity, and 0.74 MCC (see Table 3). However, for the di-methylarginine data set, the proposed model yields an accuracy of 90.40%, a specificity of 91.72%, a sensitivity of 89.16%, and an MCC of 0.80 (see Table 3). The other evaluation parameters, such as precision, f1-score, and AUC for both data sets, are shown in Fig. 5.

**Table 3:** The performance of PRMxAI on the arginine data set

Data sets	ACC (%)	SP (%)	SEN (%)	MCC
Mono-methylarginine	87.17	87.58	86.76	0.74
Di-methylarginine	90.40	91.72	89.16	0.80

Furthermore, we also used a stratified loop repeating 10-fold cross-validation 50 times [40, 41] and then average performance is calculated. The average performance of the proposed model is shown in Table 4.

**Table 4:** The performance of PRMxAI on the arginine data set by repeating the 10-fold cross-validation for 50 times

Data sets	ACC (%)	SP (%)	SEN (%)	MCC
Mono-methylarginine	84.27	84.81	83.74	0.69
Di-methylarginine	88.08	90.29	85.32	0.76

**Fig. 6:**
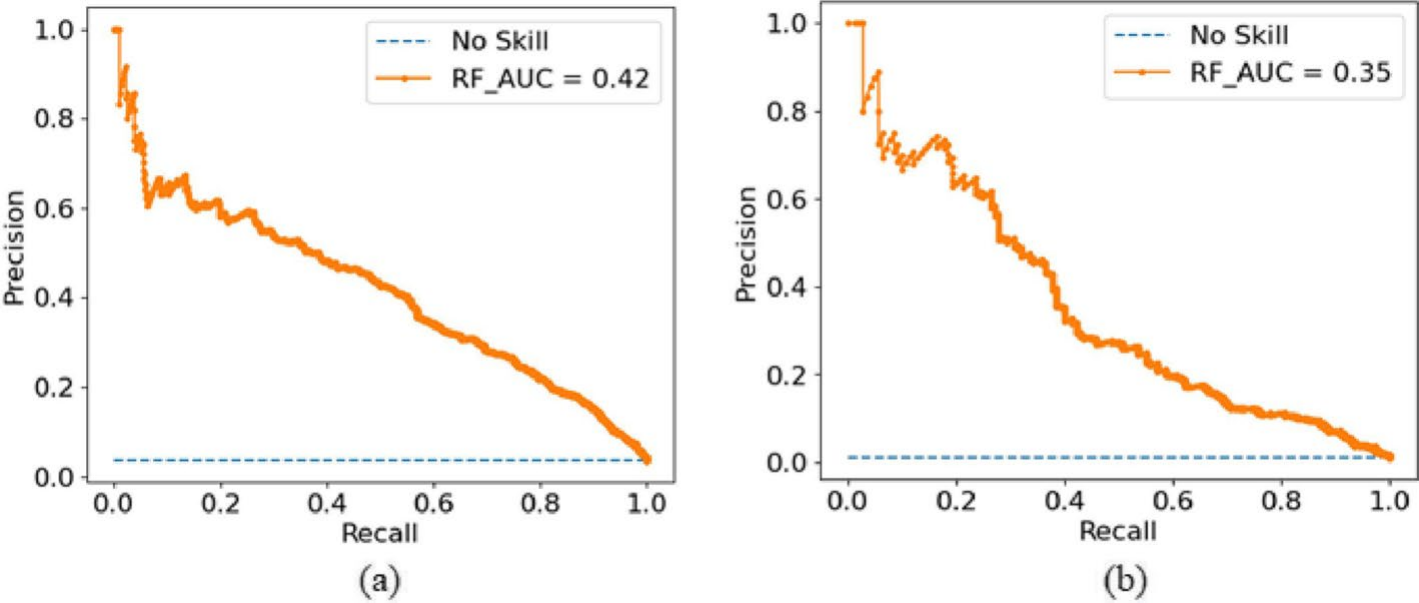
Area under the precision-recall curve for the proposed model on the imbalanced data set **a** to predict mono-methylarginine sites. **b** to predict di-methylarginine sites

We also evaluated our proposed model on the imbalanced data set. The imbalanced data set consists of 1465 mono-methylarginine, 474 di-methylarginine, and 39980 negative samples. The number of negative samples is 27 times the number of mono-methylarginine positive samples and 84 times the number of di-methylarginine positive samples. We used 70% of the data for training the model and 30% of the data for testing the proposed model. The under-sampling technique is used to balance the training data. The testing results of the proposed model for imbalanced data are shown in Table 5. The proposed model provides 85.84% accuracy, 85.94% specificity, 83.01% sensitivity, and 0.35 MCC for the mono-methylarginine data set. However, for the di-methylarginine data set, the proposed model provides an accuracy of 88.75%, a sensitivity of 86.71%, a specificity of 88.78%, and an MCC of 0.26 (see Table 5). The MCC score is low because there is a vast imbalance in the data set, so the model becomes biased towards the majority class. For the imbalanced data set, the precision-recall curve is an important measure. Figure 6 shows the area under the precision-recall curve for the imbalanced data set.

**Table 5:** The performance of PRMxAI on the imbalanced data set

Data sets	ACC (%)	SP (%)	SEN (%)	MCC
Mono-methylarginine	85.84	85.94	83.01	0.35
Di-methylarginine	88.75	88.78	86.71	0.26

## Discussion

This section discusses the effect of multiple features on the arginine data set and compares the performance of the PRMxAI against previous state-of-the-art models. This section also interprets the model outputs using the SHapley Additive exPlanation (SHAP) technique.

### Impact of various features for arginine methylated data set

The impact of various factors on the arginine methylated sites has been analyzed in this subsection. The corresponding features are taken from each protein sequence under the experiment using the same training–testing procedures. To extract feature vectors for DPC, AAC, ITB, and PP, we used 400-dimensional, 20-dimensional, 4-dimensional, and 10-dimensional feature vectors, respectively. In this research, eleven prediction models using DPC, PP, AAC, and ITB features are developed to analyze the impacts of different features. Tables 6 and 7 display the effects of the various features, i.e., DPC, PP, ITB, and AAC, on mono-methylarginine and di-methylarginine data sets, respectively.

**Table 6:** Effect of various features (DPC, PP, AAC, and ITB) for mono-methylarginine data set

Training feature	ACC (%)	SP (%)	SEN (%)	MCC
AAC	80.98	78.41	84.08	0.62
PP	73.31	77.21	70.38	0.47
DPC	73.00	66.12	90.11	0.50
ITB	50.17	55.08	55.81	0.01
AAC+PP	73.31	77.21	70.38	0.47
AAC+DPC	81.84	79.73	84.27	0.63
DPC+ITB	73.34	70.18	77.66	0.47
AAC+PP+DPC	73.31	77.21	70.38	0.47
PP+DPC+ITB	73.65	77.74	70.61	0.47
AAC+DPC+TTB	77.67	76.45	79.02	0.55
AAC+PP+DPC+ITB	87.17	87.58	86.76	0.74

**Table 7:** Effect of various features (DPC, PP, AAC, and ITB) for di-methylarginine data set

Training feature	ACC (%)	SP (%)	SEN (%)	MCC
AAC	82.27	80.72	84.00	0.64
PP	72.25	76.44	69.21	0.45
DPC	74.68	68.45	87.26	0.52
ITB	52.32	51.27	63.09	0.08
AAC+PP	72.25	76.44	69.21	0.45
AAC+DPC	83.86	83.22	84.51	0.67
DPC+ITB	69.19	68.49	69.95	0.38
AAC+PP+DPC	72.25	76.44	69.21	0.45
PP+DPC+ITB	72.89	77.74	69.47	0.46
AAC+DPC+ITB	74.57	75.60	73.63	0.49
AAC+PP+DPC+ITB	90.40	91.72	89.16	0.80

Tables 6 and 7 show that the model trained using the feature AAC outperformed other models using ITB, DPC, and PP for both problems, i.e., mono-methylarginine and di-methylarginine sites. However, the models proposed using a single feature will not be able to distinguish between methylation and non-methylated sites effectively. When the prediction model was trained with the combination of DPC and AAC (DPC+AAC) features, it performed better, as shown in Tables 6 and 7. The experimental results show that the combined features AAC+DPC+PP+ITB significantly improved the performance. This demonstrated that all four features helped differentiate between arginine methylated and non-methylated sites.

### The comparison of PRMxAI against previous predictors

This section finds the effectiveness of the proposed model PRMxAI by comparing it with the previous state-of-the-art predictors. The state-of-the-art predictors and the PRMxAI were assessed on the same data sets for an unbiased comparison. The result of the PRMxAI on the data sets utilized in [37, 39] was estimated as shown in Tables 8 and 9, respectively. For mono-methylarginine, the PRMxAI presented 87.17% accuracy, 5.07% higher than CTD-RF [37]. The accuracy of the PRMxAI for di-methylarginine was 90.40%, 7.9% higher than CTD-RF [37] (see Table 8).

**Table 8:** Comparison of the PRMxAI with CTD-RF on the data set used in CTD-RF [37] method

Prediction methods	Arginine type	ACC (%)	SEN (%)	SP (%)	MCC
CTD-RF [37]	Mono-methyl	82.1	81.9	82.4	–
	Di-methyl	82.5	82.3	82.7	–
PRMxAI (Proposed model)	Mono-methyl	87.17	86.76	87.58	0.74
	Di-methyl	90.40	89.16	91.72	0.80

To compare the performance of PRMxAI with existing models, we assessed the performance of the proposed model on the same data set used in the SSMFN method [39]. The proposed model was retrained using their training and validation data set and then tested using the independent test set to assess the proposed model. The PRMxAI was compared with BPB-PPMS [33], PMeS [42], iMethyl-PseAAC [32], MASA [31], MeMo [30], PSSMe [43], MePred-RF [35], DeepRMethylSite [38], and SSMFN [39] (see Table 9). The performances of PMeS, BPB-PPMS, MASA, MeMo, PSSMe, iMethyl-PseAAC, MePred-RF, DeepRMethylSite, and SSMFN were reported by Lumbanraja et al. [39] on the same data set. For the arginine methylation data set, the PRMxAI achieved 83.84% accuracy, 87.61% specificity, 80.76% sensitivity, and 0.68 MCC. Except for the specificity, the other three measures, i.e., accuracy, sensitivity, and MCC of the PRMxAI, were 2.69% to 27.84%, 0.76% to 68.76%, and 0.06 to 0.52 higher than the existing predictors, respectively. In conclusion, the proposed model PRMxAI performed superior to state-of-the-art classifiers, which supported the significance of AAC+PP+DPC+ITB as features for identifying arginine methylation sites.

**Table 9:** The comparison of the PRMxAI with previous predictors on the same data set used by the previous predictors [39]

Year	Author	Prediction method	Algorithm	ACC (%)	SP (%)	SEN (%)	MCC
2006	Chen et al. [30]	MeMo	SVM	68	99	38	0.46
2009	Shao et al. [33]	BPB-PPMS	SVM	56	100	12	0.25
2009	Shien et al. [31]	MASA	SVM	65	99	31	0.41
2012	Shi et al. [42]	PMeS	SVM	58	73	43	0.16
2014	Qiu et al. [32]	iMethyl-PseAAC	SVM	59	100	18	0.30
2016	Wen et al. [43]	PSSMe	SVM	72	83	60	0.44
2017	Wei et al. [35]	MePred-RF	RF	69	97	41	0.46
2020	Chaudhari et al. [38]	DeepRMethylSite	CNN, LSTM	79.42	84.47	75.08	0.60
2021	Lumbanraja et al. [39]	SSMFN	CNN, LSTM	81.15	82.40	80.00	0.62
2023	Proposed model	PRMxAI	RF	83.84	87.61	80.76	0.68

### Model interpretation using Explainable AI

Machine learning models are also known as “black box” models due to their complex internal mechanisms. One of the most challenging aspects of machine learning models has been identified as understanding the importance of every feature to the model [44]. SHAP is used to assess the contribution of each feature to the predictions of machine learning models [45]. SHAP is a global interpretation method that provides model-agnostic explainability for text, images, and tabular data. It is based on optimal Shapley values from coalitional game theory. In game theory, Shapley values allocate the value produced by a group of players fairly. The “players” in machine learning are the input features, and the “value” is the model’s output. SHAP offers local and global interpretation techniques based on aggregating the Shapley values.

**Fig. 7:**
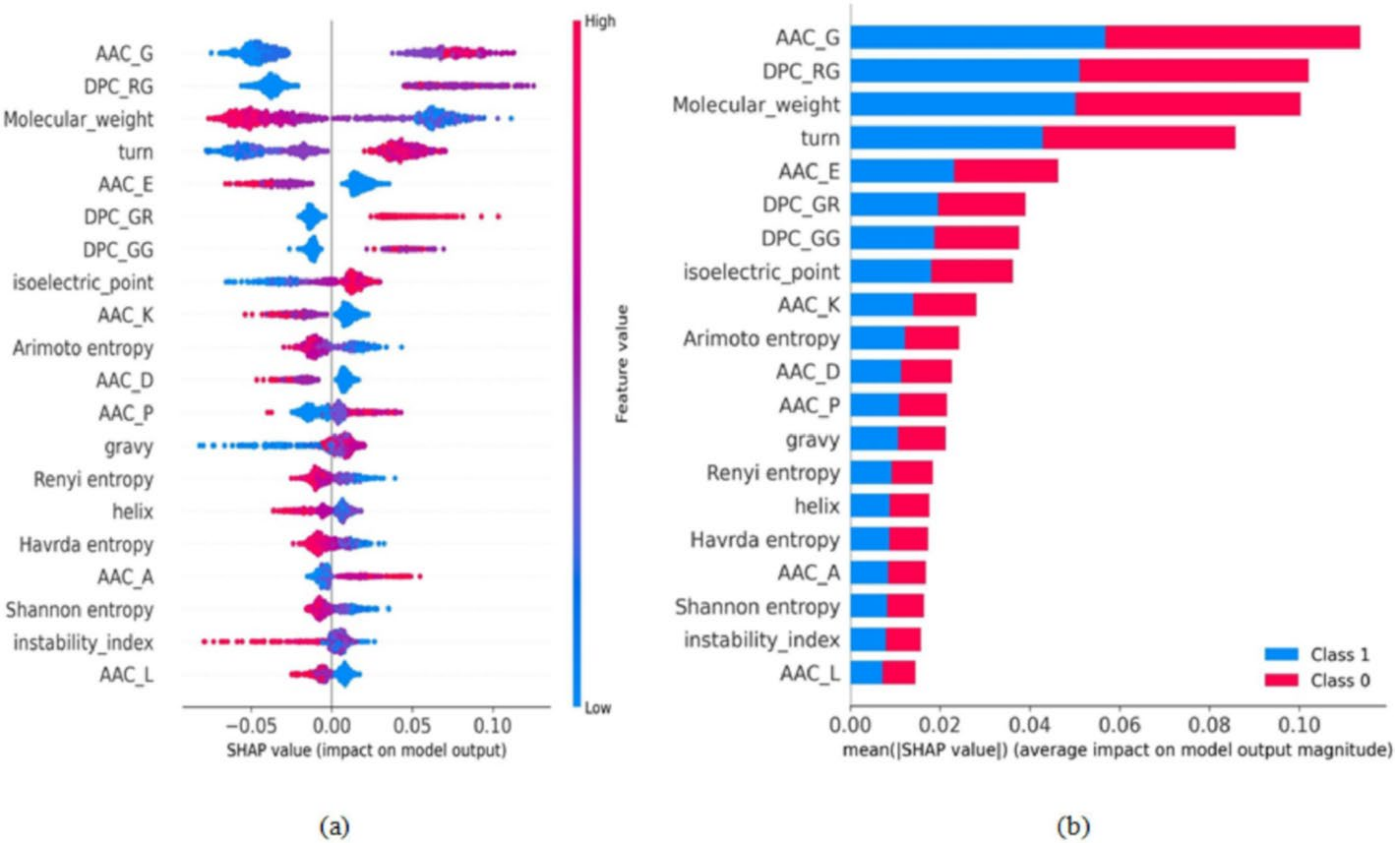
Model interpretation and feature importance for the prediction of mono-methylarginine sites. **a** Summary plot for SHAP values of top 20 features. **b** The feature’s average impact on model predictions for the top 20 features

The XAI SHAP model is used to analyze the feature importance for the proposed model by ranking them. The summary for the top 20 significant features computed using SHAP values for the mono-methylarginine sites is shown in Fig. 7a. The lowest to highest values of the features are indicated by color contrast from blue to red, as shown in Fig. 7a. From Fig. 7a, it is clear that the AAC of “glycine” amino acid and DPC of dipeptide pair “RG” has a significant impact on identifying protein methylated sites. The higher values of AAC_G and DPC_RG contribute towards the prediction of arginine methylation sites, and the lower values of AAC_G and DPC_RG contribute towards the prediction of arginine non-methylated sites. Moreover, the higher values of DPC_GR contribute to predicting arginine methylation sites. Figure 7b illustrates the top 20 features’ average impact on the proposed model output for classifying mono-methylarginine and non-methylation sites.

**Fig. 8:**
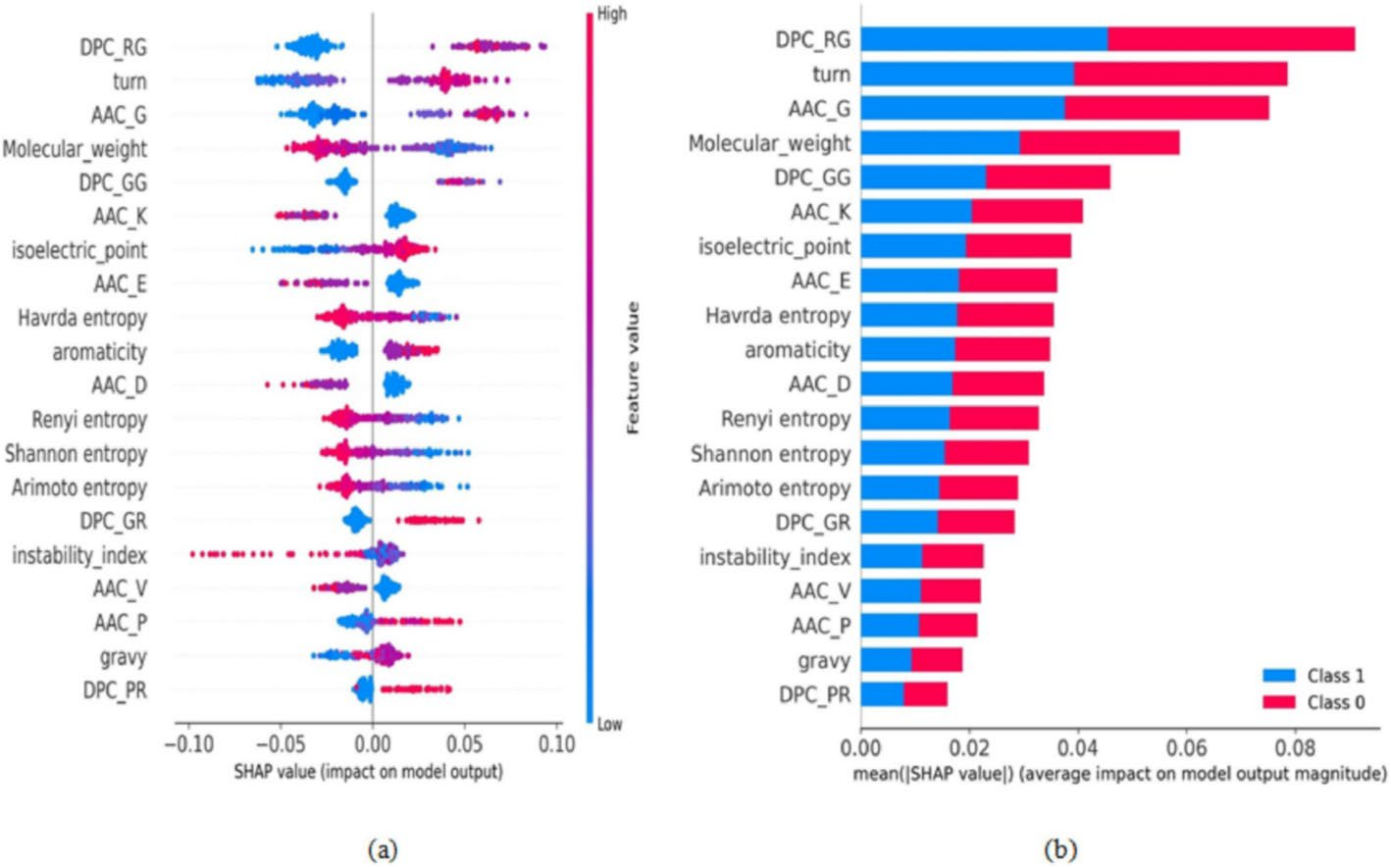
Model interpretation and feature importance for the prediction of di-methylarginine sites. **a** Summary plot for SHAP values of top 20 features. **b** The feature’s average impact on model predictions for the top 20 features

Figure 8a, b illustrate the summary plot and features’ average impact on proposed model outputs for the top 20 ranked features computed using SHAP values for di-methylarginine sites, respectively. From Fig. 8a, it can be concluded that higher values of DPC_RG, AAC_G, and turn feature descriptors contribute to predicting a positive sample. In contrast, lower values of DPC_RG, AAC_G, and turn feature descriptors contribute to predicting a negative sample. The physicochemical properties (instability index) feature descriptor significantly impact the prediction of non-methylation arginine sites. The top 20 ranked features for the identification of methylated sites consist of 4, 4, 6, and 6 feature descriptors from DPC, ITB, PP, and AAC feature extraction, respectively (see Fig. 8b). These indicate the importance of extracted features in identifying positive and negative samples.

## Conclusions

This research discusses a machine learning technique, PRMxAI, to predict arginine methylation sites and XAI SHAP to illustrate the feature importance. Each primary sequence is converted to a 434-dimensional vector by extracting informative features, i.e., AAC, DPC, ITB, and PP. These features are considered as input to the proposed RF-based model PRMxAI. The PRMxAI provided an accuracy of 87.17%, a specificity of 87.58%, a sensitivity of 86.76%, an MCC of 0.74 for the mono-methylarginine data set, and 90.40% accuracy, 89.16% sensitivity, 0.80 MCC, and 91.72% specificity for the di-methylarginine data set. The cross-validation findings indicated that the PRMxAI performed better than state-of-the-art predictors. Explainable AI is also used to analyze the importance of the features. In the future, employing fractal dimension might improve the results by detecting self-similarities within amino acid sequences [46]. The source code and data of this research is available at GitHub repository (https://github.com/Monika01p/PRMxAI_PMS).

## Methods

This section explains the data sets and features that convert protein sequences to a fixed-dimensional feature vector. A supervised learning algorithm will be used to detect methylation sites from the primary sequences.

### Data sets

Hou et al. [37] produces the arginine methylation data set. They gathered the data from the UniProtKB database (release 2020_01, www.uniprot.org) by searching the keyword ’methylarginine’ and obtained 875 proteins containing 4128 arginine sites. These 4128 peptide sequences consist of mono-methylarginine and di-methylarginine. Of 4128 peptide sequences, 3051 are mono-methylarginine, and 1077 are di-methylarginine sites. After applying CD-HIT [47] having a threshold of 0.9 to remove redundant sequences, they finally received 1465 mono-methylarginine sites and 474 di-methylarginine sites. The negative samples for mono-methylarginine and di-methylarginine are generated from 875 protein sequences where the central amino acid residue ’arginine’ is not a methylation site. We chose equal negative and positive samples for both problems (mono-methylarginine and di-methylarginine) to avoid biased results toward the class having more samples.

The data set was prepared based on Chou’s peptide strategy [48] to represent the peptide sequences in the form of arginine methylation (positive) and arginine non-methylation (negative) samples. The peptide sequence was represented as follows:

1 $$\begin{aligned} P_{\gamma }(R)= P_{-\gamma }\ldots P_{-2}P_{-1}R P_{1}P_{2}\ldots P_{\gamma } \end{aligned}$$

where *R* can be an arginine methylation or arginine non-methylation site. $P_{\gamma }$ represents the $\gamma$th upstream residue and $P_{-\gamma }$ indicates the $\gamma$th downstream residue from the center *R*. The length of the peptide sequences $P_{\gamma }(R)$ will be $2\gamma$ + 1. Hou et al. [37] considered $\gamma =5$, so the length of each peptide sequence is 11. The peptide sequences $P_{\gamma }(R)$ is considered a positive sample when the center *R* is a methylation site; otherwise, it is considered a negative sample. The positive and negative samples are merged to create the benchmark data sets for arginine methylation sites. The benchmark data sets for arginine methylation are expressed by Eq. 2.

2 $$\begin{aligned} D_{\gamma }(R)=D_{\gamma }^{+}(R)\cup D_{\gamma }^{-}(R) \end{aligned}$$

where $D_{\gamma }^{-}(R)$ and $D_{\gamma }^{+}(R)$ denote the negative and positive data set for arginine methylation sites, respectively.

### Feature representation

#### DPC

Two amino acids are combined to form a dipeptide. To create a dipeptide, two amino acids make a peptide bond. Each dipeptide’s frequency is calculated to produce a 400-dimensional vector that describes the peptide sequence [49]. The Eq. 3 is used to estimate the dipeptide composition of the peptide sequence *S* having length *l*.

3 $$\begin{aligned} D_i=\frac{n_i}{l} \end{aligned}$$

where $n_i$ is the count that tells how many times the *i*th dipeptide pair occurs within the sequence *S*.

#### AAC

Every primary sequence is made from the combinations of 20 amino acids. The occurrence of every amino acid is computed to represent the peptide sequence to the 20-D feature [50]. Suppose a peptide sequence *S* with length *k*, then Eq. 4 may be used to determine the frequency of every amino acid.

4 $$\begin{aligned} A_i = \frac{N_i}{k} \end{aligned}$$

where *k* denote the peptide sequence’s length and $N_i$ denote the count with which the *i*th amino acid occurs in the sequence. Hence, every peptide sequence is represented by:

5 $$\begin{aligned} AAC = [A_1, A_2, A_3,....., A_{20}]^T \end{aligned}$$

#### ITB

Various features from the information theory, such as Arimoto entropy (AE), Shannon entropy (SE), Havrda-Charvát entropy (HE), and Rényi entropy (RE), are computed, which are defined below.

(a) SE: An estimation of the degree of uncertainty in peptide sequences is the SE [51]. We may utilize SE to predict protein methylation sites and assess the amount of information contained within protein sequences. The below equation estimates SE:

6 $$\begin{aligned} SE = -\sum ^{20}_{i=1}{p_i} log_2{(p_i)} \end{aligned}$$

where $p_i$ specifies the occurrence of *i*th amino acids within the peptide sequence.

Relative SE measures the amino acid conservation concerning the background distribution. The Eq. 7 estimates relative SE.

7 $$\begin{aligned} Relative \; SE = \sum _{i=1}^{20} p_i\log _2\left( \frac{p_i}{p_0}\right) \end{aligned}$$

where the amount of uniformly dispersed amino acids within the sequence is indicated by $p_0$.

Whether a particular sequence is positive or negative, the information gain represents the information’s transition from the random position to the one impacted by the class. The information gain is estimated by Eq. 8.

8 $$\begin{aligned} Information \; gain = SE - Relative \; SE \end{aligned}$$

(b) HE: Havrda and Charvát [52] devised the structural entropy with degree $\alpha$, and it generalizes SE. The Eq. 9 is used to compute HE.

9 $$\begin{aligned} HE = (2^{1-\alpha }-1)^{-1} \left[ \sum _{i=1}^np_i^\alpha -1\right] \end{aligned}$$

The following equation estimates relative HE:

10 $$\begin{aligned} Relative \; HE = -(2^{1-\alpha }-1)^{-1} \left[ \sum _{i=1}^{20}\frac{p_i^\alpha }{p_0^{\alpha -1}}-1\right] \end{aligned}$$

where $$\alpha \ne 1,\alpha >0$$.

(c) RE: RE was derived by Alfred Rényi [53], and it generalizes entropies, including SE, min-entropy, and Hartley entropy. The below equation gives it:

11 $$\begin{aligned} RE = (1-\alpha )^{-1}\log \left( \sum _{i=1}^{20}p_i^\alpha \right) \end{aligned}$$

The relative RE is computed by Eq. 12.

12 $$\begin{aligned} Relative \; RE = (1-\alpha )^{-1}\log \left( \sum _{i=1}^{20}\frac{p_i^\alpha }{p_0^{\alpha -1}}\right) \end{aligned}$$

where $$\alpha \ne 1,\alpha >0$$.

(d) AE: Arimoto proposed the generalized entropy having a real parameter [54]. The Eq. 13 is used to computing AE.

13 $$\begin{aligned} AE = (2^{\alpha -1}-1)^{-1}\left[ \left( \sum _{i=1}^{20} p_i^{1/\alpha }\right) ^\alpha -1\right] \end{aligned}$$

The below equation gives relative AE:

14 $$\begin{aligned} Relative \; AE = -(2^{\alpha -1}-1)^{-1}\left[ \left( \sum _{i=1}^{20} \frac{p_i^{1/\alpha }}{p_0^{1/(\alpha -1)}}\right) ^\alpha -1\right] \end{aligned}$$

where $$\alpha \ne 1,\alpha >0$$.

#### PP

Different PP features were estimated utilizing the ProtParam web-server [55], such as isoelectric point, extinction coefficients (EX), instability index, molecular weight, aromaticity, helix, sheet, turn, and grand average of hydropathy (GRAVY).

(a) EX: The EX shows how much light a protein takes at various wavelengths. When purifying a protein, it is helpful to calculate this coefficient by utilizing ProtParam server [55]. The molar EX of the protein is estimated from the AAC. With the help of the molar EX of cystine, tyrosine, and tryptophan, the EX of protein in water is estimated by Eq. 15.

15 $$\begin{aligned} EX = ME(cy)*n(cy)+ME(tr)*n(tr)+ME(ty)*n(ty) \end{aligned}$$

where *ME*(*cy*), *ME*(*ty*), and *ME*(*tr*) denote the molar EX of cystine, tyrosine, and tryptophan, respectively. Whereas *n*(*cy*), *n*(*tr*), and *n*(*ty*) denote the count of cystine, tryptophan, and tyrosine residues per molecule, respectively.

(b) Instability index: The instability index determines whether a protein is stable in the test tube. A protein possessing an instability index value of more than 40 is unstable, and one with less than 40 is stable. There are 400 dipeptide pairs, and [56] set a dipeptide instability weight value (DIWV) for every dipeptide. The Eq. 16 was utilized to calculate the instability index.

16 $$\begin{aligned} Instability \; index = \frac{10}{L}\sum ^{L-1}_{i=1}DIWV([A_i A_{i+1}]) \end{aligned}$$

where DIWV([$A_i A_{i+1}$]) specifies the instability weight value starting at *i*th index and *L* specifies the peptide sequence length.

(c) GRAVY: The value of GRAVY for an amino acid sequence was calculated as the summation of hydropathy values [57] for all amino acids, divided by the length of the protein sequence. The online web server ProtParam [55] is used to estimate it.

### RF algorithm

Leo Breiman first presented the RF classifier [58]. It depends upon ensemble learning and consists of a collection of DT from the subset of features via a random feature selection approach. The count of features in every tree is influenced by various aspects, including dependency, the strength of the classifier, and generalization error. RF was applied in various computational biology applications, i.e., protein-protein interaction, DNA-binding proteins identification [59], and protein fold prediction [60]. This paper implements the proposed PRMxAI using Intel Xeon(R) CPU E5-1650 v4 @ 3.60GHz with six cores and 12 processors, Python 3.9.12, and Keras 2.12.0 on the Windows operating system.

Let $X = {x_1, x_2, x_3,...., x_N}$ denote the set of instances, *A* represents the attribute, and $X_v$ is subset of *X* with $A = v$. The RF algorithm is stated by Algorithm 1. 
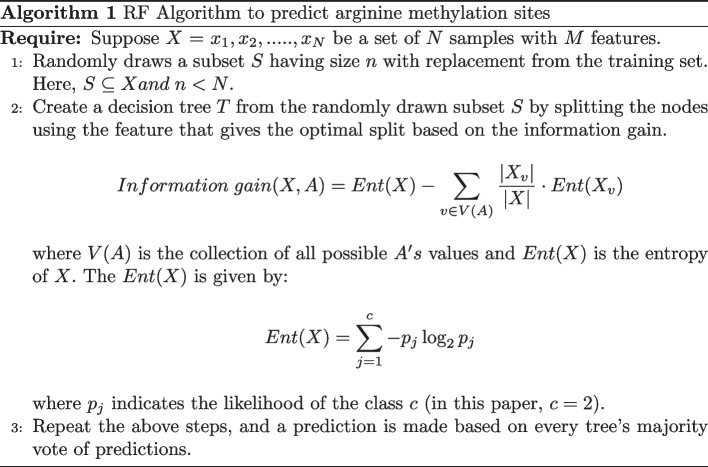


#### Model training

Machine learning algorithms need feature extraction for finding useful and discriminative patterns from the primary sequences. Four feature representation methods were used to convert each primary sequence into numerical representation for training the models. The extracted features are then given as input to the machine learning algorithms. This paper used the RF algorithm as the base classifier for training the model and generating predictions for protein methylation sites. The optimized hyperparameters for the RF algorithm are given in Table 10. We used a grid search method to find the optimum values of hyperparameters for the RF model [44, 61, 62]. We tried different numbers of trees, such as 10, 50, 100, 150, 200, 250, 300, 350, 400, 450, and 500, for selecting the optimal number of trees in the forest. Different depths ranging from 10 to 100 with a gap of 10 were chosen to find the maximum depth of a tree in RF. Other hyperparameters, such as $min\_samples\_split$, $max\_features$, $max\_samples$ are obtained using grid search.

**Table 10:** Optimum values of hyperparameters used for the RF algorithm

Parameters	Optimum value
n_estimators	250
max_depth	40
max_features	log2
min_samples_split	3
max_samples	1.0

### Framework of the proposed model

**Fig. 9:**
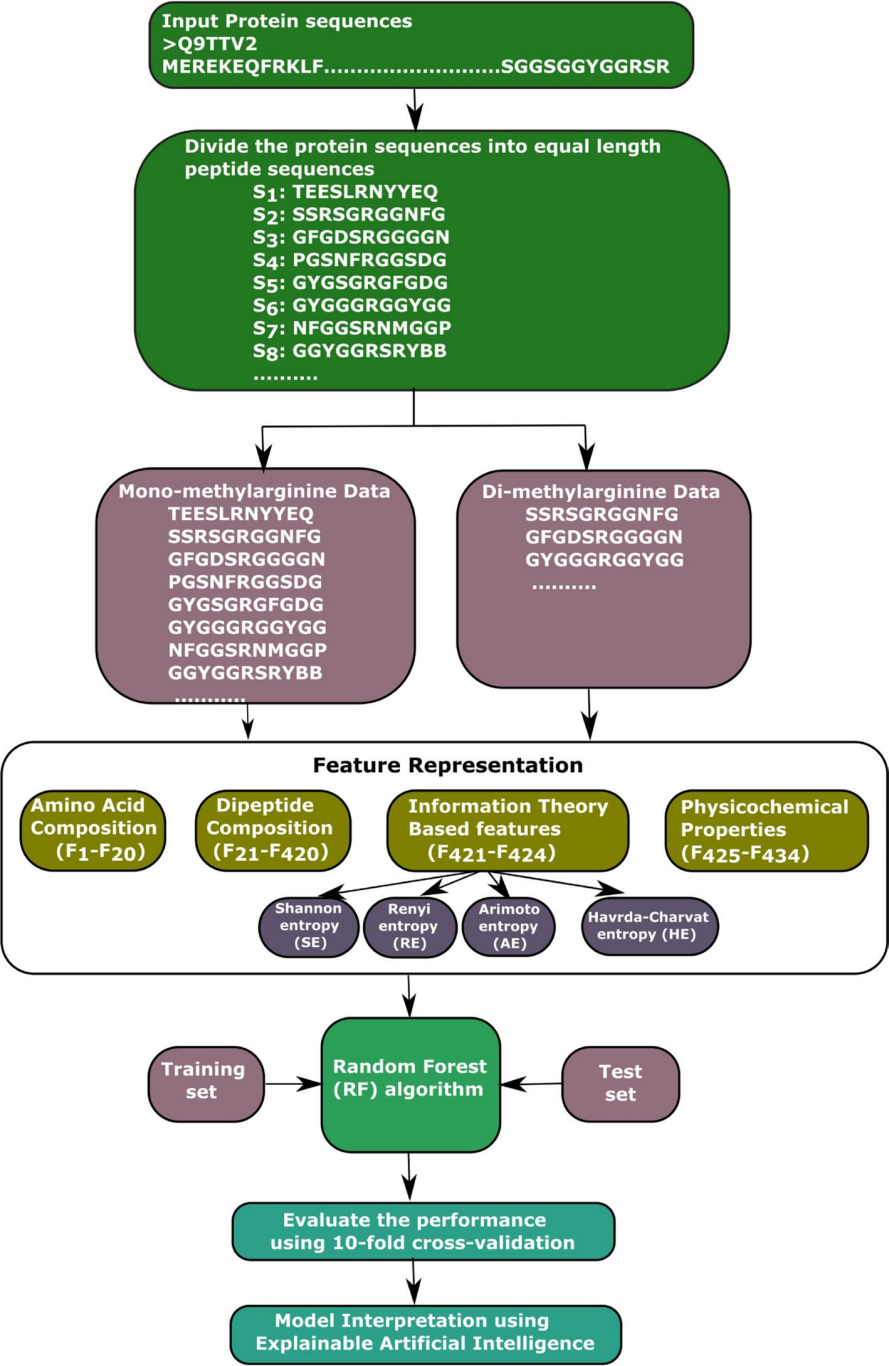
Flow diagram of the proposed PRMxAI for predicting mono-methylarginine and di-methylarginine sites in protein sequences. $F_1-F_{20}$ describe the feature vector generated by amino acid composition; $F_{21}-F_{420}$ represents the feature vector obtained using dipeptide composition; $F_{421}-F_{424}$ describe the feature vector given by information theory-based features; and $F_{425}-F_{434}$ provide the feature vector produced by physicochemical properties

A supervised machine learning model is proposed in this research to recognize arginine methylation sites from primary sequences. First, using a sliding window, each amino acid sequence is divided into peptide sequences having the same length. Then, choose the peptide sequences with R as their center while rejecting the others. The next step is to characterize each peptide sequence by a 434-dimensional feature vector by extracting features, such as dipeptide composition, physicochemical properties, amino acid composition, and information theory-based features (Arimoto, Havrda-Charvat, Renyi, and Shannon entropy), from the amino acid sequences. Then, an RF classifier is utilized for training the model and making predictions for arginine methylation sites. The performance of the proposed PRMxAI is evaluated by employing 10-fold cross-validation. The flow diagram of the proposed model is shown in Fig. 9.

### Evaluation metrics

The effectiveness of the PRMxAI was analyzed using 10-fold cross-validations. The data set was partitioned into ten roughly equal-size subsets. Then, nine subsets were utilized for training, and the unused subset was utilized for evaluating the model. This method was performed ten times, utilizing a different subset for testing to create ten models. The final performance was estimated using the average of all these ten models.

The evaluation metrics, such as ACC, SEN, SP, precision, f1-score, and MCC, are defined by the below equations:

17 $$\begin{aligned} SP= {} \frac{TN}{TN+FP} \end{aligned}$$

18 $$\begin{aligned} ACC= {} \frac{TN+TP}{TP+FN+TN+FP} \end{aligned}$$

19 $$\begin{aligned} SEN= {} \frac{TP}{TP+FN} \end{aligned}$$

20 $$\begin{aligned} MCC= {} \frac{TP \times TN-FP \times FN}{\sqrt{(FP+TP)(FN+TP)(TN+FP)(FN+TN)}} \end{aligned}$$

21 $$\begin{aligned} Precision= {} \frac{TP}{TP+FP} \end{aligned}$$

22 $$\begin{aligned} f1-score= {} 2* \frac{Precision*SEN}{Precision+SEN} \end{aligned}$$

The corresponding counts for true positives, false positives, true negatives, and false negatives are denoted by *TP*, *FP*, *TN*, and *FN*, respectively.

## Data Availability

The data set for this research is taken from UniProtKB database (release 2020_01, www.uniprot.org).

## Abbreviations

AAC: Amino acid composition
ACC: Accuracy
AE: Arimoto entropy
AUC: Area under ROC curve
CNN: Convolutional neural network
DPC: Dipeptide composition
DT: Decision tree
HE: Havrda–Charvát entropy
ITB: Information theory-based features
KNN: K-neaest neighbors
LSTM: Long short term memory
MCC: Matthew’s correlation coefficient
NB: Naive Bayes
PP: Physicochemical properties
PRTM: Protein arginine methyltransferases
PTMs: Post-translational modifications
RE: Rényi entropy
ROC: Receiver operating characteristics
RF: Random forest
SE: Shannon entropy
SEN: Sensitivity
SHAP: SHapley Additive exPlanation
SP: Specificity
SVM: Support vector machines
XAI: Explainable artificial intelligence
